# Our use, misuse, and abandonment of a concept: Whither habitat?

**DOI:** 10.1002/ece3.3812

**Published:** 2018-04-02

**Authors:** David Anthony Kirk, Allysia C. Park, Adam C. Smith, Briar J. Howes, Brigid K. Prouse, Naschelly G. Kyssa, Elizabeth N. Fairhurst, Kent A. Prior

**Affiliations:** ^1^ Aquila Conservation & Environment Consulting Ottawa ON Canada; ^2^ School of Science and the Environment Memorial University Corner Brook NL Canada; ^3^ Canadian Wildlife Service Environment and Climate Change Canada Ottawa ON Canada; ^4^ Natural Resource Conservation, Parks Canada Gatineau QC Canada; ^5^ Department of Biology University of Ottawa Ottawa ON Canada; ^6^ Department of Biology Dalhousie University Halifax NS Canada; ^7^ Present address: Grenfell Campus Memorial University Corner Brook NL Canada; ^8^ Present address: Laurentian University Sudbury ON Canada; ^9^ Present address: Toronto Wildlife Centre North York ON Canada; ^10^ Present address: Malvern Veterinary Hospital Scarborough ON Canada; ^11^ Present address: Envirowest Consultants Inc. Port Coquitlam BC Canada

**Keywords:** conservation implications, critical habitat, habitat, habitat‐related terms, misuse, operational terminology, standardization

## Abstract

The foundational concept of habitat lies at the very root of the entire science of ecology, but inaccurate use of the term compromises scientific rigor and communication among scientists and nonscientists. In 1997, Hall, Krausman & Morrison showed that ‘habitat’ was used correctly in only 55% of articles. We ask whether use of the term has been more accurate since their plea for standardization and whether use varies across the broader range of journals and taxa in the contemporary literature (1998–2012). We searched contemporary literature for ‘habitat’ and habitat‐related terms, ranking usage as either correct or incorrect, following a simplified version of Hall et al.'s definitions. We used generalized linear models to compare use of the term in contemporary literature with the papers reviewed by Hall et al. and to test the effects of taxa, journal impact in the contemporary articles and effects due to authors that cited Hall et al. Use of the term ‘habitat’ has not improved; it was still only used correctly about 55% of the time in the contemporary data. Proportionately more correct uses occurred in articles that focused on animals compared to ones that included plants, and papers that cited Hall et al. did use the term correctly more often. However, journal impact had no effect. Some habitat terms are more likely to be misused than others, notably ‘habitat type’, usually used to refer to vegetation type, and ‘suitable habitat’ or ‘unsuitable habitat’, which are either redundant or nonsensical by definition. Inaccurate and inconsistent use of the term can lead to (1) misinterpretation of scientific findings; (2) inefficient use of conservation resources; (3) ineffective identification and prioritization of protected areas; (4) limited comparability among studies; and (5) miscommunication of science‐based findings. Correct usage would improve communication with scientists and nonscientists, thereby benefiting conservation efforts, and ecology as a science.

Habitat is “the resources and conditions present in an area that produce occupancy – including survival and reproduction – by a given organism. Habitat is organism‐specific; it relates the presence of a species, population, or individual (animal or plant) to an area's physical and biological characteristics. Habitat implies more than vegetation or vegetation structure; it is the sum of the specific resources that are needed by organisms.”

(Hall, Krausman & Morrison, 1997)

## 1 INTRODUCTION

Since 1970, it is estimated that on average vertebrate species’ populations (including mammals, birds, reptiles, amphibians, and fish) have declined by 52% as a result of human activities (Living Planet Index; WWF International, 2014). The primary driver causing these species’ population declines and extinctions is loss of habitat (Brook, Sodhi, & Bradshaw, 2008; Kerr & Deguise, 2004; Millennium Ecosystem Assessment, 2005; Venter et al., 2006). Loss of habitat could interact in complex, unforeseen ways with the looming global threat of climate change (Mantyka‐Pringle et al., 2015). But despite the recognition that habitat plays a key role in stemming global population declines, even the primary ecological literature may not support a consistent definition for the term (Hall, Krausman & Morrison, 1997; Guthery & Strickland, 2015; Mathewson & Morrison, 2015; see Krausman & Morrison, 2016).

‘Habitat’ is used to describe virtually every kind of location occupied by organisms—from the small‐scale microcosm habitat of hot spring pools which are the home of the Banff Spring Snail (*Physella johnsoni*—Lepitzki & Pacas, 2010) to the vast plains of the Serengeti Mara Ecosystem and other African savannas, where migratory herds of Wildebeest (*Connochaetes taurinus*) and Elephants (*Loxodonta africana*) track new green vegetation (Bohrer, Beck, Ngene, Skidmore, & Douglas‐Hamilton, 2014; Boone, Thirgood, & Hopcraft, 2006; Jarman & Sinclair, 1979). In some cases, correct use and identification of habitat could have direct implications for human life. For example, identifying temporal and spatial habitat for Great White Sharks (*Carcharodon carcharias*) can help mitigate threats to humans in inshore waters (Kock et al., 2013) and similarly delineating Tiger *(Panthera tigris*) habitat in the Sundarban in India and Bangladesh can avoid conflicts with people (Naha et al., 2016).

Used today almost ubiquitously in the ecological and conservation literature, the word ‘habitat’ is a Panchreston problem, similar to that identified for another commonly used ecological term, ‘fragmentation’ (Bunnell, 1999; Fischer & Lindenmayer, 2007). The Panchreston problem refers to “an explanation or theory used in such a variety of ways as to become meaningless.” According to a search on Google Scholar, the term ‘habitat’ has been deployed in at least 2.4 million publications. Moreover, habitat‐related terminology pervades the scientific literature (Krausman, 1999) and includes a whole gamut of terms—among them; habitat type, habitat use, suitable habitat, habitat requirements, habitat fragmentation, and habitat heterogeneity. Ironically, if used correctly, most of these habitat‐related terms are self‐contradictory or oxymorons, for example, ‘habitat heterogeneity,’ ‘unsuitable habitat,’ and ‘habitat type’ (Mathewson & Morrison, 2015; Krausman & Morrison, 2016; see Box 1).

Box 1 **Definitions for habitat‐related terms (from Krausman & Morrison (2016)—used with permission)**

1

**Table:** 

Habitat—The resources and conditions present in an area that produce occupancy, which may include survival and reproduction by a given organism. Habitat is organism‐specific and is more than vegetation or vegetation structure. Thus, suitable habitat is redundant and unsuitable habitat is a misnomer; if it was unsuitable, it would not be habitat! Neither term should be used. Instead reference unsuitable areas or unsuitable vegetation types. Habitat abundance—The amount of habitat available regardless of its availability to animals. Because researchers can measure abundance, using this term is more accurate than using habitat availability. Habitat availability—The accessibility and procurability of physical and biological components of a habitat by animals. Habitat quality—The ability of the environment to provide conditions appropriate for individual and population persistence. Quality should be based on the demographics of the population and not necessarily numbers alone. Quality is an outcome (e.g., survival and productivity) and is not a user‐defined inherent property of a location. For example, Hall et al. (1997) suggested low habitat quality represents the resources available for survival, medium habitat quality represents resources available for survival and reproduction, and high‐quality habitat includes resources available for population persistence. These are critical distinctions because a geographic location (e.g., study area) could fluctuate from year‐to‐year in some critical resource (e.g., berry or insect production) yet retain the same basic vegetation composition and structure. Thus, habitat quality could vary from year to year. Habitat type—This term refers only to the type of vegetation association in an area or to the potential of vegetation to reach a specified climax stage (Daubenmire, 1968:72–73). Habitat type describes vegetation, not an animal species’ habitat. To describe vegetation in an animal's habitat, use vegetation types and associations to avoid confusion with habitat type. Habitat use—The way an animal uses or consumes a collection of physical and biological resources. Habitat selection—Habitat selection is a hierarchical process that an animal uses to choose habitat components (Hutto, 1985; Johnson, 1980)

‘Habitat’ has been similarly defined by several authors: Hall et al. (1997) described it as “The resources and conditions present in an area that produce occupancy – including survival and reproduction – by a given organism” (Hall et al., 1997, 175). Garshelis (2000, 112) defined it as “…. the type of place where an animal normally lives or, more specifically, the collection of resources and conditions necessary for its occupancy.” More recently, Morrison (2009, p. 61) described habitat as “an area with a combination of resources (e.g., food, cover, water) and environmental conditions (e.g., temperature, precipitation, presence or absence of predators and competitors) that promotes occupancy by individuals of a given species (or population) and allows those individuals to survive and reproduce.” Hall et al. (1997) argued that “Habitat is organism‐specific; it relates the presence of a species, population, or individual (animal or plant) to an area's physical and biological characteristics. Habitat implies more than vegetation or vegetation structure; it is the sum of the specific resources that are needed by organisms.” Moreover, habitat is invariably measured by ecologists from a human perspective, which may be quite different to what organisms perceive. Here, we provide support and endorsement for the definition of ‘habitat’ used in the original paper by Hall et al. (1997). Twenty years ago, Hall et al. (1997) found that only nine of 50 (18%) of the articles they reviewed defined and used the term ‘habitat’ and habitat‐related terms consistently according to category 1 (strictest definition) in their standard terminology (see Methods). They found that use of the term ‘habitat’ varied for two main reasons: (1) Habitat relations varied according to spatial extent, with few authors documenting the scale of their investigations or limiting their discussions to the scale of their work (the implication being that results could not be extrapolated to other situations and contexts); (2) many authors used the term ‘habitat’ to refer to vegetation association or vegetation type, but ‘habitat’ is not synonymous with these terms.

So why is it important that the term ‘habitat’ be used correctly today, and why should scientists care? First, inaccurate and inconsistent use of the term can lead to misinterpretation of scientific findings (Herrando‐Pérez, Brook, & Bradshaw, 2014). Given that habitat loss is the primary driver of species’ population declines and extinctions, identifying and correctly defining habitat for organisms is necessary for its protection, management, and restoration. Not only do we need to evaluate and quantify the effects of human activities on species’ habitat, but we also must weigh the relative roles of species’ habitat loss compared to other threats, such as land cover fragmentation, invasive organisms, climate change, contaminants, or overexploitation (Wilcove, Rothstein, Dubow, Phillips, & Losos, 1998; Evans et al., 2011; Lesbarrères et al., 2014). Furthermore, we should measure the cumulative effects of those threats (e.g., Halpern & Fujita, 2013).

Second, and related to the above, incorrectly defining ‘habitat’ could lead to inefficient use of conservation resources. In a world where resources for conservation planning and biodiversity conservation are always limited, cost is a critical consideration (Carwardine et al., 2008; Daily & Ellison, 2002; Keith, Vardon, Stein, Stein, & Lindenmayer, 2017; Naidoo et al., 2006). Thus, we need to efficiently define and protect habitat in the face of limited conservation resources and increasing development pressures.

Third, misusing the term ‘habitat’ could lead to ineffective identification and prioritization of protected areas. Understanding the features and parameters that comprise habitat for individual species and how environmental changes impact those factors is part of this identification and prioritization. We need to be able to define and map habitat for as many species as possible, particularly for species at risk (Reed et al., 2006).

Armed with this information, we can then make predictions and, in turn, take management and conservation action. Advances in technology have meant that our ability to map habitat for species is greatly enhanced. Perhaps the potential to misuse the term is even greater now that such mapping has become so widely adopted; the temptation is to call GIS‐derived features, such as forest and wetland, ‘habitat’ (e.g., Evans, Costa, Tomas, & Camilo, 2014), whereas in fact ‘land cover’ would be a more accurate term. Another issue is that habitat by definition is species‐specific, but there has been a trend in ecology toward viewing ecosystems as a whole, rather than individual species, and perhaps this is why use of the term has become even more muddled. Examples are the use of the habitat‐related terms ‘habitat type’ and ‘habitat heterogeneity’ where ‘habitat’ is used in a generic sense.

Fourth, misusing the term ‘habitat’ means that there is potential for comparability among studies to become limited and, by extension, conducting meta‐analyses and complementarity to identify generic insights into species–habitat relationships.

Fifth and finally, we need to be able to communicate the concept of habitat effectively among scientists and between scientists and nonscientists. This means we need to define the term ‘habitat’ appropriately and adhere to this definition consistently (Guthery & Strickland, 2015). Hall et al. (1997) concluded that the inappropriate use of the word ‘habitat’ hinders communication with other scientists, managers, and the public. This ambiguity surrounding the use of the term can have widespread repercussions. For example, in Canada, there is a legal requirement to identify critical habitat for species at risk—defined as the habitat necessary for the survival or recovery of a listed endangered, threatened, or extirpated species in Schedule 1 of the Species at Risk Act (SARA, 2002).

The need for operationalizing scientific terms has never been more important, and yet according to Mathewson and Morrison (2015), little has changed in this regard over the last 50 years. While others have recently researched use of the term (e.g., Guthery & Strickland, 2015) and reiterated pleas for standard terminology (Krausman & Morrison, 2016), ours is the first systematic and quantitative study of how use of the term has changed over time. In this study, we searched the contemporary literature to compare our findings with Hall et al.'s results.

We conducted similar searches to those conducted by Hall et al., with three important modifications: (1) We asked whether analyzing articles in a wider range of journals than those used by Hall et al. would yield different conclusions. (2) We investigated a broader range of taxa to see whether there were taxon‐related differences in use of the term ‘habitat.’ (3) We asked whether it is the use of the term ‘habitat’ itself (the umbrella term) that is incorrect, or whether the problem lies in misuse of the numerous habitat‐related terms. We tested the effect of period (articles analyzed by Hall et al. vs. contemporary articles published since their publication), on use of the term. Within the contemporary literature (post‐1997), we tested whether the correct use of the term ‘habitat’ differed between articles that dealt with animals (mammal, bird, reptile, amphibian, and fish) versus those that included discussions of habitat use by plants. Finally, we assessed whether articles that specifically cited Hall et al. (1997) were more likely to use the term correctly than those that did not on the assumption that the authors of these articles would be more careful or consistent in their use of the term than those authors not citing Hall et al. (1997).

Like Hall et al. (1997), we believe that the need to operationalize^1^ the term is important, while also recognizing the limitations of the concept (Mathewson & Morrison, 2015). We agree with Peters (1991) that without a clear, operational definition, users of terms are able to develop their own interpretations of what they mean. And there is no justification for the sloppy use of terms in ecology (Morrison & Hall, 2002). Articles in peer‐reviewed journals play a pivotal role in adoption of science‐based evidence for practical conservation management (Ewen, Adams, & Renwick, 2013), which makes it critical to operationalize our terms, including use of the word ‘habitat.’

## 2 MATERIALS AND METHODS

### 2.1 Sampling articles that use the term ‘habitat’

Hall et al. (1997) examined the use of the term ‘habitat’ in 50 articles from 11 journals and several scientific books and technical manuals between 1980 and 1994. They chose these journals and books because they represented (1) current (as of 1994) important wildlife publications (e.g., Wildlife Techniques Manuals, 4th and 5th editions—Schemnitz, 1980; Bookhout, 1994) and (2) bird–habitat and mammalian–habitat relationships (Table 1 in their paper, p. 175).

#### 2.1.1 Post‐1997 survey of contemporary articles

To objectively review the scientific literature, we used a search engine (Web of Science) for the time period post‐Hall et al.'s publication (1998 to 2012, hereafter ‘post‐1997’ or ‘contemporary’). We deliberately omitted the 4 years post‐1994 (the endpoint year examined by Hall et al.) because we also wanted to see whether the publication of Hall et al.'s, paper influenced usage of the term. This enabled us to compare their data (Table 2 in Hall et al.**,** 1997, p. 176) and our own data, and to determine whether the use of the term ‘habitat’ had improved since Hall et al.'s plea for standardization.

We carried out exploratory analyses to determine how many articles we could find within journals containing the word ‘habitat.’ In the first phase of investigations, we replicated Hall et al.'s methods using the same nine journals (Table S1) and 22 habitat‐related search terms (Table S2) for the post‐1997 period. This initial search yielded 2,385 articles containing the word ‘habitat.’ We randomly selected 50 of these articles (the same number analyzed in Hall et al., 1997) in equal proportion from each journal for analysis. This represented 2% of the total search results.

For the second phase of investigations, we expanded the journal selection to include those that were not sampled in phase 1, but had a high impact factor (in the Web of Science category of ‘Ecology’), and that included studies on nonmammal vertebrate taxa. We included journals with a high impact factor because these may be more influential in how the term was used and theoretically should set a higher standard for correct usage. Many new ecology and conservation journals have been launched since Hall et al.'s publication, and we wanted to make sure we sampled a broad range of these journals; for example, 17 of the 50 journals we chose were first published in or after 1994 (Tables S1 and S2). We initially chose the top 30 journals (out of 129) that had the highest impact factor. Of those, two journals were not available in the Web of Science database, an additional two did not contain articles with ‘habitat’ in the title, and five were included in phase 1. We therefore chose the nine journals with the next highest impact factor to retain a total of 30 journals. A search of these 30 journals employing the same search terms used in phase 1 and Hall et al. yielded a total of 8,858 articles containing the word ‘habitat.’ To sample the same proportion of articles as phase 1, our goal was to analyze at least 177 articles in equal proportion from each of the journals chosen. In total, we analyzed 185 articles for phase 2.

The above exploratory analyses provided us with a starting point for which journals to include in the analysis. We selected a total of 50 journals that we felt were representative and where we expected the term ‘habitat’ to be used. Of these, 32 were selected (Table S1) and were all listed among the top 76 ecological and conservation journals based on their 2014 ISI ranking and Google ranks (Bradshaw, 2014). We included an additional 18 journals to cover some specific taxonomic groups not included in the former list (e.g., that specialized on fish) and also to include more specialized or regional journals not listed by Bradshaw (2014) but nonetheless contained articles describing the habitat associations of different taxa (Table S2). We reviewed 235 articles in total for phases 1 and 2, in addition to 80 articles that cited Hall et al. directly (see below) making a grand total of 315 articles.

#### 2.1.2 Analysis of habitat‐related terms

We examined each article to see how the term ‘habitat’ was used and then identified all habitat‐related terms using the search tool in Adobe Acrobat. While each term (‘habitat’ or habitat‐related term) was assessed based on its use throughout the article, we focused particular attention on the initial few uses of the word within each article, assuming that the first few use(s) of the word would reflect how the term is applied for the remainder of the article and should therefore be consistent thereafter. To start rating how the term is used, we began our search in the Introduction since abstract word limits prevent authors from providing detailed definitions. In the Introduction, we expected authors to elaborate on and explain their meaning of the term so readers can understand the context for the remainder of the article. We scored the usage of the term ‘habitat’ according to the scoring system below.

On a scale of 1–4, we indicated whether the usage of the term ‘habitat’ was (1) consistently and correctly used throughout the article with an explicit definition similar or identical to the one given by Hall et al., 1997; (2) used acceptably and consistently but with no definition; (3) used inconsistently (weak usage) with no definition; or (4) used incorrectly—no definition supplied and ‘habitat’ was confused with other terms (e.g., ‘vegetation type’ and ‘land cover’). We then read through the article for other habitat‐related terms and scored them in the same way. Multiple uses of different terms were common in the articles we reviewed. For example, ‘habitat’ may be used as well as ‘habitat use’ or ‘suitable habitat.’

Some habitat‐related terms we found were not listed by Hall et al. For example, the term ‘habitat fragmentation’ has been used extensively post‐1997. We deemed it important to include such terms in our statistical analyses. However, we simplified many terms in the models as they essentially had the same meaning (see column 2 in Table S3). Of the terms in Table S3, 12 were used more than 10 times in the contemporary data. We combined all other habitat‐related terms (many of which had <5 occurrences in our sample) into the category of ‘other’ (Table S3). Note that some of these habitat‐related terms are, strictly speaking, nonsensical (e.g., ‘suitable habitat’ and ‘unsuitable habitat’).

### 2.2 Statistical analysis

#### 2.2.1 Comparing all contemporary data (post‐1997) with Hall et al. (1997)

For all analyses, we pooled categories 1 and 2 into a ‘correct’ category and categories 3 and 4 into an ‘incorrect’ category. Note that Hall et al. did not provide detailed definitions for each habitat‐related term, because they did not consider the use of most of these terms as valid.

We used a generalized linear model (GLM) with a binomial error (logistic regression) to test the effect of phase (Hall et al. vs. contemporary data) on use of the term. In the same model, we included an effect of term, to test for differences in the usage among the habitat‐related terms (including ‘habitat’). From this analysis, we excluded all contemporary articles that considered the habitat of plants, to align the taxa between the contemporary data and Hall et al.'s original data, which did not include plant species’ habitat.

#### 2.2.2 Effect of taxa and journal on contemporary usage

To test whether usage differed between articles that dealt with different taxa, we fit an additional binomial GLM to the contemporary data that included an effect of term and a categorical effect of taxa to compare usage between articles that considered only the habitat of animals (including mammal, bird, reptile, amphibian, and fish) and those that also considered the habitat of plants. To test whether journal ranking influenced usage, we fit an additional binomial GLM to the contemporary data that included an effect of term and a categorical effect of journal rank to compare usage between articles from journals with higher ISI impact factors and Google ranks (top 20 journals in Table S3) and all other journals. Our rationale was that journals with higher ISI impact factors and Google ranks would be harder to publish in and perhaps have more critical or rigorous review processes to maintain extremely high standards, and by implication should have more correct uses of terms. All models were run in the R statistical package (R Core Development Team 2013).

#### 2.2.3 Did authors citing Hall et al. (1997) use the term more correctly?

Finally, we analyzed a subset of articles that specifically cited Hall et al. (1997). We predicted that authors who cited Hall et al. (1997) directly should be using the term ‘habitat’ correctly, at least in relation to authors who did not cite Hall et al. To do this, we added this term to the binomial model above to test the effect. We found a total of 290 articles that cited Hall et al. (1997) by the year 2016 and we reviewed a random sample of 80 of these articles, which we felt were representative of the literature.

## 3 RESULTS

### 3.1 Has use of the term ‘habitat’ improved since Hall et al. (1997)?

**Figure 1:**
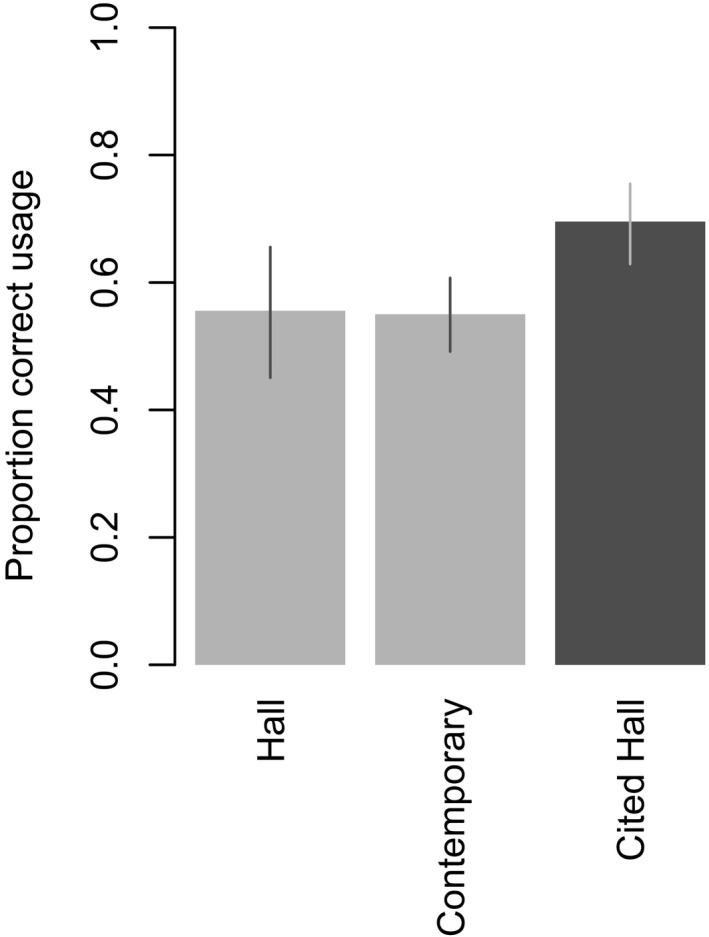
Predicted proportion of correct uses of the term ‘habitat’ in articles reviewed in Hall et al. (1997); compared to contemporary articles and articles citing Hall et al. Note: The predicted differences between the Hall et al. (1997) articles and the contemporary articles are identical for all other habitat‐related terms and so are not shown here. Error bars indicate the 95% confidence intervals

We found no significant difference between the correct use of ‘habitat’ in the articles reviewed by Hall et al. and the contemporary articles, after accounting for the varying use of habitat‐related terms (Table S4). For example, our model results showed that the word ‘habitat’ alone was used correctly on approximately 55% of occasions in both the articles reviewed by Hall et al. and the contemporary articles. (Note that because we combined correct use in the strict sense with correct use with no definition our figures differ from those reported by Hall et al.; see Figure 1.) In fact, correct use of the term ‘habitat’ alone (correct use in the strictest sense) was actually higher in the earlier than the later period (5 of 41 articles or 10.9% vs. 4 of 231 articles or 1.7%).

### 3.2 How does usage vary among habitat‐related terms?

**Figure 2:**
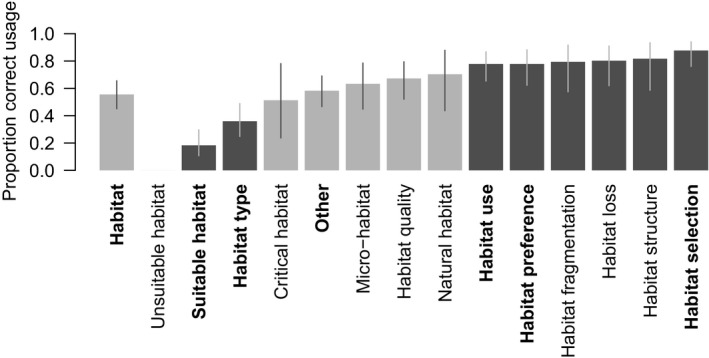
The predicted proportion of correct uses of habitat‐related terms, for contemporary data (since the publication of Hall et al. (1997)), excluding the articles that cited cited Hall et al. (1997). Note: Predictions for the Hall et al. (1997). articles are essentially identical and are not provided here. The terms in bold on the horizontal axis were relatively common in the Hall et al. articles (used in five or more articles). The bars that are darker gray indicated terms for which the correct usage was significantly different from the correct usage for the main term ‘habitat.’ Error bars indicate the 95% confidence intervals of the predictions for each term, and their colors vary only to provide a visual contrast with the main bar

Some habitat‐related terms were used incorrectly more often than ‘habitat’ on its own and others were more often used correctly (Figure 2, and Table S4). This is largely because many of these habitat‐related terms can never be used correctly (e.g., ‘suitable habitat’ and ‘unsuitable habitat’) as they are nonsensical (Krausman & Morrison, 2016; Mathewson & Morrison, 2015). The terms ‘suitable habitat’ and ‘habitat type’ were significantly less likely to be used correctly. Although not strictly statistically significant—the model estimates an infinite variance for a term with no variation in its usage (Table S4)—in fact, ‘unsuitable habitat’ is self‐contradictory, and so cannot be used correctly. By contrast, the terms ‘habitat loss,’ ‘habitat preference,’ ‘habitat use,’ ‘habitat structure,’ and ‘habitat selection’ were significantly more likely to be used correctly than ‘habitat’ on its own (Table S4). The usage of the remaining habitat‐related terms was not significantly different than that of the term ‘habitat’ alone.

### 3.3 What is the influence of taxa and journal ranking in the contemporary data?

**Figure 3:**
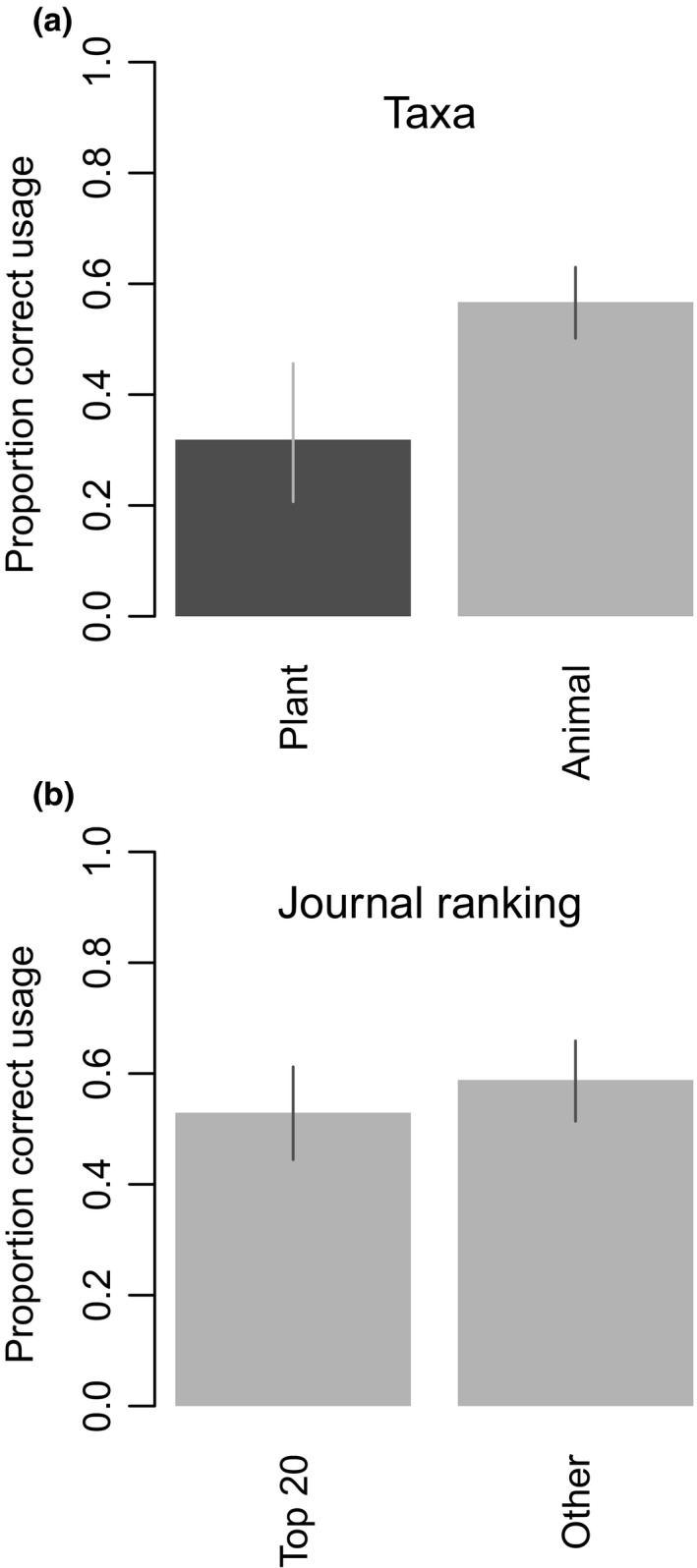
Predicted proportion of correct uses of the term ‘habitat‘ in contemporary articles by primary taxa (a: articles including plant habitat—plant—compared to those that only include animal habitat—animal and grouped by journal ranking (b: articles from top‐ranked journals compared to those from all other journals). Note: The predicted differences between taxa and journal rank are identical for all other habitat‐related terms, and so are not shown here. Error bars indicate the 95% confidence intervals

In the contemporary articles, those that included plants were significantly less likely to use habitat terms correctly than articles that only included animals (Figure 3a and Table S5), after controlling for the use of habitat‐related terms. Because the Hall et al. articles did not include any that focused on plant habitat and usage in these articles was significantly worse, we removed the plant articles from the contemporary data for all other analyses. We found no significant difference in usage of the term ‘habitat’ between the top 20 ISI/Google‐ranked journals and all other journals (Table S6; Figure 3b), after controlling for the use of habitat‐related terms. Indeed, the direction of the very small difference was actually contrary to our predictions; the top‐ranked journals did not have better or poorer usage than other journals.

### 3.4 Do authors who cite Hall et al. (1997). use the term ‘habitat’ correctly?

The Web of Science search revealed a total of 290 citations of Hall et al. (1997) and 31 citations of Morrison and Hall (2002; a companion paper to Hall et al., 1997). Our model results indicated that authors who cited Hall et al. did indeed use the term correctly more often than those who did not (approximately 70% were correct; Figure 1).

## 4 DISCUSSION

Our results demonstrated that the term ‘habitat’ has not been used more correctly post‐1997 than in the previous period analyzed by Hall et al. (pre‐1995). ‘Habitat’ was used correctly in only approximately half the articles we reviewed both prior to 1995 and post‐1997. We conclude that Hall et al.'s plea has been largely ignored by most researchers or at least forgotten in the intervening period.

We also found that some habitat‐related terms were much less likely to be used correctly than others. Certain terms appeared to be traps for misuse—in particular ‘habitat type,’ ‘suitable habitat,’ and ‘unsuitable habitat.’ ‘Habitat type’ was used incorrectly in most cases because the authors were referring to land cover types or plant assemblages. The problem with ‘unsuitable habitat’ is that it is a contradiction in terms, and the term ‘suitable habitat,’ of course implies the existence of ‘unsuitable habitat.’ ‘Habitat‘ ‘is’ a binary term; either a particular space is habitat for a given species, or it is not (Krausman & Morrison, 2016; Mathewson & Morrison, 2015). On the other hand, terms such as ‘habitat structure’ and ‘habitat selection’ were used correctly in more than 80% of articles. Note that Mathewson and Morrison (2015) recommended using only four habitat‐related terms (‘habitat use‘, ‘habitat preference‘, ‘habitat selection‘, and ‘habitat quality‘), though Krausman and Morrison (2016) list seven—see Box 1.

Our secondary findings were, first, articles that focused on animals were more likely to employ correct usage of the term ‘habitat’ than articles that focused on both animals and plants. This suggested that there were taxon‐related differences in the use of the term. Second, correct usage was not proportionately higher in the top 20 ISI/Google‐ranked journals than all other journals and in fact was slightly less. Finally, perhaps not surprisingly, articles that directly cited Hall et al. (1997) used ‘habitat’ correctly more often than those that did not, however, even some articles that cited Hall et al. (1997) included incorrect uses of the term.

### 4.1 Consequences of misuse

Operationalizing the terms we use as ecologists will minimize confusion (Peters, 1991) and is not only essential if we want to be taken seriously but also critical to the precision and efficacy of our scientific advice. Perhaps the greatest source of confusion is that ‘habitat’ is frequently used at two different scales—at the level of an individual species and at the level of species assemblages (i.e., generic usage). The debate on habitat loss versus fragmentation is also largely based on a generic definition of habitat applicable to species assemblages. For example, Fahrig's (2013) habitat amount hypothesis is proposed to explain variation in species richness of particular assemblages, though it can be used to predict the occurrence of individual species. To partly circumvent this problem, Rountree and Able (2007) suggested that two terms could be used: ‘Organismal habitat type’ to refer to individual species’ habitat in the sense of Hall et al. (1997) and ‘Ecological habitat type’ to refer to community‐level description (analogous to ‘vegetation type’). However, we would argue that ‘ecological vegetation type‘ or ‘biotope’ would be a more appropriate description here, rather than ‘habitat type’. For example, to describe the home of a suite of species (e.g., coral reefs), the more appropriate terminology would be ‘ecotype‘ or ‘biome‘ (Mathewson & Morrison, 2015). In regard to use of the term ‘fragmentation,’ studies should refer to species‐specific habitat fragmentation, while our suggestions for generic usage could be ‘land cover fragmentation’ or ‘vegetation fragmentation.’

Misusing the word ‘habitat’ could have several consequences, some general and others more specific. We outline five specific consequences of misuse of the word below:

1. *Misinterpretation of scientific findings:* Researchers can misinterpret each other's scientific findings—or the misuse of the term could lead to misunderstandings in other concepts such as metapopulation theory (Mathewson & Morrison, 2015). Perhaps the worst offender and one for which most (many) of us are guilty of is the use of ‘habitat’ to describe vegetation type or land cover (Hall et al., 1997; Krausman, 1999). By extension, not all native vegetation is habitat for some species (e.g., it may not have the structural complexity required for habitat), and some species can survive in human‐modified landscapes. Paul Krausman (1999) cites an example which may be familiar to many scientists; when he hears someone say, “This is great wildlife habitat,” he feels as if he has “walked into a brick wall.” The question is habitat for which species? Species have different habitat requirements and what constitutes habitat for one species does not provide the resources for reproduction and/or survival for another. For example, in the Canadian boreal forest, through disturbance and succession, a given location can offer habitat for a wide variety of bird species over time (Kirk, Diamond, Hobson, & Smith, 1996; Schieck & Song, 2002). Natural disturbance regimes play a major role in altering not only land cover but also landscape structure and, in turn, species’ occurrences (see Drapeau, Villard, Leduc, & Hannon, 2016). Another example is farmland in North America and Europe which provides habitat through multiple land cover types, heterogeneity, and food sources for many farmland bird species (Lindsay et al., 2013; Martin et al., 2012; Vickery, Bradbury, Henderson, Eaton, & Grice, 2004).
2. *Inefficient use of conservation resources:* Debate over what does and does not constitute habitat within or among ecologists, land managers, and industry representatives is a waste of critical resources that could be directed elsewhere. This debate can often lead to delayed conservation action and can increase legal scrutiny and risk with respect to habitat protection. For example, the identification of critical habitat for the northern and southern resident Killer Whales (*Orcinus orca*) in British Columbia (see Fisheries and Oceans Canada, 2011, 2017) was delayed by years because of disagreements between government officials and members of the species’ recovery team over the definition of (critical) habitat. The disagreement included whether habitat should include nongeophysical descriptions or elements (e.g., acoustic underwater disturbance, prey availability, and specifically Chinook Salmon *Oncorhynchus tshawytscha*). Eventually, nongovernment organizations successfully filed a lawsuit to force the Government of Canada to include nongeophysical components of critical habitat (Federal Court, 2010). The lack of clarity surrounding the term ‘critical habitat’ resulted in disagreements and disputes that delayed the finalization of the Recovery Strategy by 5 years, while threats increased and the species continued to decline. The Killer Whale critical habitat issue is a good example of many of the problems and misconceptions of the concept including the idea that habitat is a single‐dimension, spatially‐delineated area (see Mathewson & Morrison, 2015). In fact, Mathewson and Morrison (2015) suggest that the term ‘critical habitat’ is an oversimplification of the habitat concept and could potentially result in inappropriate management for wildlife and misuse of resources. In recovery planning documents, ‘critical habitat’ is referring to a population and not individuals and perhaps may be a valid term. While it may be valid, it can be very challenging to identify; it could be described as the subset of all habitat needed to support a minimum viable population of a species and will always have a spatial component. Thus there is likely to be some amount or particular area of habitat that, if lost, could lead to extirpation of a population, whereas other areas of habitat could be lost without harming population persistence. This view can be problematic because it portrays habitat as a spatially defined area, yet many of the components of habitat can be nonspatial. Obviously, the idea of protecting habitat for species at risk should not be in question, but the terminology associated with this idea may require further discussion. Perhaps a more appropriate and correct term for a species’ critical habitat would be ‘high priority management area,’ though this may not carry as much weight as ‘critical habitat,’ which is the core of species at risk protection in the Species at Risk Act in Canada.
3. *Ineffective identification and prioritization of protected areas:* Misuse of the term ‘habitat’ can also hinder efforts to identify and prioritize protected areas to conserve biodiversity (Moilanen, Wilson, & Possingam, 2009). Protected area planning requires identification of habitats for multiple species and by extension, communities (e.g., hotspots for biodiversity). Not all areas are equally important for wildlife conservation, although they may have differing degrees of habitat quality for individual species. We need to know how much habitat a species needs at any given location to maintain a viable population (e.g., Fahrig, 2001), although consideration of its configuration across the landscape can still be important in some situations and below certain thresholds (the *fragmentation threshold hypothesis*; Betts, Forbes, & Diamond, 2007; Betts et al., 2014; Villard & Metzger, 2014; Jackson & Fahrig, 2016).
4. *Miscommunication and comparability across areas or studies:* An increasing trend in the ecological literature is to perform meta‐analysis combining the results of multiple studies in an overall modeling paradigm. In cases where ‘habitat’ is used as a metric, it is usually being used in the generic sense rather than in terms of its original meaning which is species‐specific (e.g., Watling et al., 2011). Fischer and Lindenmayer (2007) refer to a meta‐analysis by Debinski and Holt (2000) which produced inconsistent results (contradicting theory) because some authors were referring to habitat as native vegetation or wilderness while others were referring to habitat specific to individual species (i.e., the correct definition according to Hall et al., 1997 and Mathewson & Morrison, 2015). By extension, not all native vegetation is habitat for some species (e.g., it may not have the structural complexity required for habitat), and some species can survive in human‐modified landscapes. If we refer to ‘habitat’ as all types of land cover, regardless of whether or not they provide resources for individual species, then this is misuse of the term. Generic forms of use of the term (‘habitat amount’, ‘habitat fragmentation’, and ‘habitat heterogeneity’) are really adulterations of its original meaning. Patches of forest in a fragmented landscape may provide habitat for multiple species, but unless species‐specific habitats have been identified and delineated, they should be referred to as ‘forest patches’ and not ‘habitat patches.’
5. *Communication among scientists and between scientists and nonscientists:* In some respects, this negative consequence of misuse overlaps the first point (misrepresentation of scientific results among scientists). It differs from the first point in that one of the most important practical dangers of misusing the term ‘habitat’ is how the concept is communicated to nonscientists, particularly in relation to land use conflicts. How can ecologists and conservation biologists be taken seriously by industry and legal representatives if they cannot operationalize their own key concepts? On a practical note, we also need to know how to reconcile the habitat needs of multiple species so that we can target high‐priority areas for conservation and to enable countries to meet their international targets for representation and complementarity. Systematic conservation planning tools such as Marxan and Zonation, for example, can help in this regard and use algorithms to spatially prioritize protected area networks for representation and biodiversity features (Sarkar et al., 2006; Moilanen & Ball, 2009). They provide a rigorous and data‐driven framework incorporating information from habitat models or ecological niche models (Elith & Leathwick, 2009). For example, in Virunga National Park, Democratic Republic of Congo, oil exploration has now put into question over 40% of the park's land and the future viability of the Mountain Gorilla (*Gorilla berengei berengei*) and it is possible that the park boundaries may be reduced to accommodate oil exploration. Without a common understanding of what the term ‘habitat’ means and constitutes for this species—and others—we cannot know what is needed by the gorillas (e.g., using species distribution models—Kayijamahe, 2008) and we cannot conserve their habitat and the habitat required by other species within the national park (see also Jones et al., 2016). Also, protecting habitat alone for species is not in itself an effective conservation strategy without considering the spatial prioritization of threats and the cost‐effectiveness of addressing and mitigating those threats (Carwardine et al., 2012).

### 4.2 Recommendations and conclusions

We recommend that authors:

1. Refer to Hall et al. (1997), Mathewson and Morrison (2015), Krausman and Morrison (2016) and this paper to check that they are using the term ‘habitat’ correctly in their manuscripts. Consistency in use is key, as many authors switch meanings within the same article (Mathewson & Morrison, 2015). Guthery and Strickland (2015) suggested that authors define what they mean by the word ‘habitat’ and then apply the term consistently. However, the problem with this approach is that authors could create their own definitions and simply continue to define the term improperly (M. Morrison, 2016, personal communication).
2. As part of operationalizing use of the term ‘habitat’, it is important for authors to define the components of habitat for a species in a specifically quantitative way (i.e., average tree height, grass cover, and patch size). This will inherently improve the clarity of scientific findings and to facilitate comparability among studies.
3. Recognize the pitfalls of using some habitat‐related terms. The worst offenders, ‘suitable habitat’ or ‘unsuitable habitat,’ should never be used (as the first contains redundant terms and the latter is an oxymoron). This includes making sure that ‘habitat’ is not used interchangeably with ‘vegetation association’ or ‘substrate association.’ Mathewson and Morrison (2015) recommend using only four habitat terms: ‘habitat use’, ‘habitat selection’, ‘habitat preference’, and ‘habitat quality’. Krausman and Morrison (2016) recently expanded these terms to seven (see Box 1).
4. Exercise extreme caution when using the word ‘habitat’ in a legal context (e.g., ‘critical habitat’) to ensure that use is not ambiguous.

Defining ‘habitat’ correctly and consistently is the first step to ensuring the better protection of biodiversity. Given the drastic and unprecedented magnitude of decreases in biodiversity, one of the most pressing needs is to identify and prioritize habitat for as many living species as possible. This is not a task that can be achieved by a single ecologist and for the results obtained by multiple ecologists to be useful, they must all be founded on the correct definition of the term. Only then will results be able to be combined by overlaying maps and conducting meta‐analyses. Overlaying multiple species' habitats will allow coinciding threats to be identified and mitigated (e.g., James, Ottensmeyer, & Myers, 2005) and cumulative effects to be assessed. It will also enable us to restore habitat that has been lost in many areas (Morrison, 2009). Another important consideration is that many species have conflicting habitat requirements and so we need to set population targets or goals for habitat thresholds using optimization or other types of models (e.g., Holzkämper, Lausch, & Seppelt, 2006; Thogmartin, Crimmins, & Pearce, 2014; Westphal, Field, & Possingham, 2007). If we do not know the habitat parameters for as many species as possible, both in terms of amount and spatial arrangement (including the size, amount, and functional connectivity of areas), then we cannot protect and restore habitat to prevent the inexorable creep of human development that threatens the future of the earth's biota (e.g., the Bornean Orangutan *Pongo pygmaeus*; Gregory et al., 2012).

However, it is important to recognize that merely defining ‘habitat’ consistently and correctly will not eliminate disagreement about what constitutes habitat for a species. In the example of the Killer Whales discussed above, defining ‘habitat’ consistently would not eliminate the possibility that different parties would disagree about whether to explicitly include whales' food source. Inevitably, different researchers will understand and model the ecology of species differently. Further, one should be acutely aware that the habitat of a given species may vary geographically and, to some extent, through time, especially given climate change (e.g., Ball et al., 2016; Wiens, Rotenberry, & Van Horne, 1987); thus findings from different years or different areas may provide different (but equally valid) results. According to Mathewson and Morrison (2015), to accurately denote a species’ habitat, one must obtain demographic information on vital rates and long‐term persistence *in space and time* to determine habitat quality. While this is an admirable goal, it is extremely challenging to conduct such intensive and expensive investigations for multiple species. And even if this was attainable, we cannot manage landscapes and ecosystems for a single species to the exclusion of all others. A way around this dilemma is to use complementarity to combine single species and ecosystem approaches as suggested by Fischer and Lindenmayer (2007).

We suggest that abandoning metrics such as abundance and occupancy as measures of species’ habitat use as implied by some (e.g., Mathewson & Morrison, 2015) may be throwing out the proverbial baby with the bathwater. Precisely defining ‘habitat’ and making management decisions based on this for each and every species is not an achievable goal because costs would be exorbitant and at the expense of gathering information on other species within a region. The important point is that once ‘habitat’ is identified (from occupancy by an organism), then habitat quality can be measured using several metrics. Thus there is not a ‘habitat gradient’ but rather a gradient in habitat quality. On the other hand, if we prioritize habitat studies for individual species and address key knowledge gaps, then we can use complementarity to marry single species and ecosystem approaches (see Fischer & Lindenmayer, 2007 for examples).

As Mathewson and Morrison state (2015, p. 7), “In a world where land management and conservation are the pinnacle goals for most ecological studies, we are faced with a desire to provide general, holistic management prescriptions and conservation initiatives.” We think this is absolutely true and would further suggest that studies of land cover (previously ‘habitat’), together with species‐specific habitat studies, are vital to inform conservation and management actions (Fischer & Lindenmayer, 2007). We agree with Mathewson and Morrison (2015) that, “we must start with a basic set of terms that *recognizes the limitations* (our italics) that the basic concept of habitat can provide.” Striving for complementarity between the great potential of geographic information systems (recognizing their limits and assumptions) and quantitative information on habitat for individual species may be a path forward for improving our use of the term and conserving species diversity.

## CONFLICT OF INTEREST

None declared.

## AUTHOR CONTRIBUTION

DAK wrote the manuscript, incorporated revisions, ran preliminary statistics, managed the project, and compiled the literature; AP and EF performed the first round of literature searches; ACS conducted the final statistical modeling and commented on drafts; BH coordinated the literature reviews and made editorial contributions to drafts; BP and NK reviewed 100 articles as part of an undergraduate university project; and KAP conceived, helped with developing methodology, and helped secure funding for the study. All authors critically reviewed drafts and gave final approval for publication.

## ENDNOTE

1 To operationalize is to set something up so that it can be measured.
