# Supplementary material for: Our use, misuse, and abandonment of a concept: Whither habitat?
Source: Ecol Evol. 2018 Apr 2;8(8):4197–208. doi: 10.1002/ece3.3812 (PMC5916312; doi:10.1002/ece3.3812)
Supplement: Supplementary file 1 [file ECE3-8-4197-s001.docx]

**Supplementary Material**

Supplementary Table 1. Conservation or ecological journals included in study - journals listed in order of ranking (average Google and ISI impact ranks + coefficient of variation; Bradshaw 2014). **Bolded** journals were included in Hall *et al.* 1997; *italicized* journals are post-1995.

| Journal | Year of first publication | ISI ranks (2014) | Average rank + CV |
| --- | --- | --- | --- |
| Science | 1880 | 31.477 | 2 |
| *Ecology Letters* | 1998 | 13.042 | 7.783 |
| *Global Change Biology* | 1995 | 8.224 | 11.684 |
| Molecular Ecology | 1992 | 5.840 | 15.189 |
| *Frontiers in Ecology and the Environment* | 2003 | 8.412 | 15.819 |
| Proceedings of the Royal Society B: Biological Sciences | 1905 | 5.292 | 17.499 |
| **Ecology** | 1920 | 5.000 | 19.899 |
| *Journal of Ecology* | 1998 | 5.694 | 21.73 |
| Bioscience | 1964 | 5.439 | 22.064 |
| *Global Ecology and Biogeography* | 1999 | 7.242 | 23.034 |
| Journal of Applied Ecology | 1964 | 4.754 | 23.307 |
| Annual Review of Ecology, Evolution and Systematics | 1970 | 10.977 | 23.922 |
| **Conservation Biology** | 1987 | 4.320 | 23.951 |
| Biological Conservation | 1968 | 4.036 | 25.048 |
| **Ecological Applications** | 1991 | 4.126 | 25.453 |
| Functional Ecology | 1987 | 4.857 | 25.583 |
| Journal of Biogeography | 1974 | 4.969 | 27.052 |
| Journal of Animal Ecology | 1932 | 4.726 | 28.051 |
| *Diversity and Distributions* | 1998 | 5.469 | 28.922 |
| **Ecological Monographs** | 1931 | 7.107 | 31.73 |
| Ecography | 1978 | 4.207 | 36.157 |
| Forest Ecology Management | 1977 | 2.667 | 36.55 |
| Oikos | 1949 | 3.559 | 39 |
| Oecologia | 1968 | 3.248 | 39.625 |
| Landscape Ecology | 1988 | 3.574 | 41.619 |
| *Biological Invasions* | 1999 | 2.716 | 45.609 |
| Biodiversity and Conservation | 1992 | 2.065 | 53.187 |
| *Basic and Applied Ecology* | 2000 | 2.389 | 57.025 |
| Environmental Conservation | 1974 | 2.320 | 60.582 |
| **Journal of Wildlife Management** | 1937 | 1.611 | 63.18 |
| Wildlife Research | 1956 | 1.487 | 70.570 |
| Bird Conservation International | 1991 | 1.554 | 72.51 |

Supplementary Table 2. Ecology and conservation journals included in search not included in Bradshaw’s (2014) ranking (all contemporary data) - journals listed alphabetically. **Bolded** journals were included in Hall *et al.* 1997; *italicized* journals are post-1995.

| Journal | Year of first publication | ISI ranks (2014) | Average rank + CV |
| --- | --- | --- | --- |
| Aquatic Ecology | 1968 | 1.456 | n/a |
| *Behavioral Ecology* | 1999 | 3.157 | n/a |
| *Biodiversity* | 2000 |  | n/a |
| *Ecosystems* | 1998 | 3.531 | n/a |
| Ecotoxicology | 1992 | 2.500 | n/a |
| **Environmental Management** | 1977 | 1.648 | n/a |
| *Evolution* | 2000 | 4.659 | n/a |
| Evolutionary Ecology | 1987 | 2.372 | n/a |
| *Fish and Fisheries* | 2000 | 8.755 | n/a |
| Heredity | 1947 | 3.804 | n/a |
| *Herpetological Conservation and Biology* | 2006 | 0.653 | n/a |
| Journal of Evolutionary Biology | 1988 | 3.483 | n/a |
| **Journal of Mammalogy** | 1919 | 2.225 | n/a |
| *Mammalian Biology* | 2002? | 1.337 | n/a |
| *Northeastern Naturalist* | 1994? | 0.361 | n/a |
| *Perspectives in Plant Ecology, Evolution and Systematics* | 1998 | 3.324 | n/a |
| **Southwestern Naturalist** | 1956 | 0.207 | n/a |
| **Wildlife Society Bulletin** | 1973 | 1.27? | n/a |

SupplementaryTable 3. Simplified list of habitat-related terms extracted from our review of the literature (22 terms analyzed by Hall *et al*. 1997 are bolded in ‘Original term’ column)

| Original term | Simplified term |
| --- | --- |
| **Habitat** | Habitat |
| **Critical habitat** | Critical habitat |
| Habitat fragmentation | Habitat fragmentation |
| Habitat destruction | Habitat loss |
| **Habitat loss** | Habitat loss |
| **Habitat preference** | Habitat preference |
| Habitat preferences | Habitat preference |
| **Habitat quality** | Habitat quality |
| **Habitat selection** | Habitat selection |
| Habitat selection theory | Habitat selection |
| **Habitat structure** | Habitat structure |
| **Habitat type** | Habitat type |
| **Habitat types** | Habitat type |
| Habitat usage | Habitat use |
| **Habitat use** | Habitat use |
| **Habitat utilization** | Habitat use |
| Habitat-use | Habitat use |
| **Macrohabitat** | Micro-habitat |
| Mesohabitat | Micro-habitat |
| **Microhabitats** | Micro-habitat |
| **Natural habitat** | Natural habitat |
| Natural habitats | Natural habitat |
| **Abandoned habitat** | Other |
| Adequate habitat | Other |
| Appropriate habitat | Other |
| **Availability of habitat** | Other |
| Available habitat | Other |
| Habitat relationship | Other |
| Habitat abundance | Other |
| Habitat accommodation | Other |
| Habitat alteration | Other |
| Habitat area | Other |
| **Habitat availability** | Other |
| Habitat change | Other |
| Habitat changes | Other |
| Habitat characteristics | Other |
| Habitat coefficient | Other |
| Habitat complexity | Other |
| Habitat conservation | Other |
| Habitat covariates | Other |
| Habitat degradation | Other |
| Habitat degregation | Other |
| Habitat distribution | Other |
| Habitat disturbance | Other |
| Habitat diversity | Other |
| Habitat dynamics | Other |
| Habitat element | Other |
| Habitat heterogeneity | Other |
| Habitat linkages | Other |
| Habitat manipulation | Other |
| Habitat matrix | Other |
| Habitat modification | Other |
| Habitat mosaics | Other |
| Habitat patches | Other |
| Habitat predictors | Other |
| Habitat relationships | Other |
| Habitat requirement | Other |
| Habitat specialist | Other |
| Habitat specialists | Other |
| Habitat specialization | Other |
| Habitat specific | Other |
| **Habitat suitability** | Other |
| Habitat suitability models | Other |
| Habitat sustainability | Other |
| Habitat switch/shift | Other |
| Habitat unsuitability | Other |
| Habitat variables | Other |
| High quality habitat | Other |
| **High value habitat** | Other |
| **Less desirable habitat** | Other |
| Microhabitat selection | Other |
| Microhabitat use | Other |
| Nesting habitat | Other |
| Nonhabitat | Other |
| Non-habitat | Other |
| **Occupied habitat** | Other |
| Optimal habitat | Other |
| Optimum habitat | Other |
| Past habitat | Other |
| Productive habitat | Other |
| Quality habitat | Other |
| Secondary-habitat | Other |
| Suboptimal habitat | Other |
| Total potential habitat | Other |
| Unfavourable habitat | Other |
| Unlikely habitat | Other |
| Unoccupied habitat | Other |
| Unurbanized habitats | Other |
| **Unused habitat** | Other |
| Vacant habitat | Other |
| Vertical habitat | Other |
| Habitat suitability | Suitable habitat |
| **Suitable habitat** | Suitable habitat |
| Suitable habitats | Suitable habitat |
| Uninhabitable habitat | Unsuitable habitat |
| Unsuitable habitat | Unsuitable habitat |

Supplementary Table 4. Parameter estimates from a binomial generalized linear model fitted to compare correct use of habitat and related terms between articles reviewed by Hall *et al*. 1997 and contemporary articles. Note: the correct use of habitat in articles reviewed in Hall et al. 1997 is the reference category in this analysis. Significance *** P < 0.001, ** P < 0.01, * P < 0.05, n = P < 0.1.

|  | Estimate | Std. Error | z value | Pr(>\|z\|) |  |
| --- | --- | --- | --- | --- | --- |
| (Intercept) | 0.223 | 0.215 | 1.036 | 0.300 |  |
| Contemporary | -0.022 | 0.219 | -0.099 | 0.921 |  |
| Cited Hall et al. | 0.603 | 0.232 | 2.605 | 0.009 | ** |
| Other terms | 0.111 | 0.169 | 0.658 | 0.510 |  |
| Critical habitat | -0.171 | 0.604 | -0.283 | 0.777 |  |
| Habitat selection | 1.741 | 0.391 | 4.457 | <0.000 | *** |
| Habitat quality | 0.496 | 0.277 | 1.791 | 0.073 |  |
| Habitat use | 1.034 | 0.286 | 3.611 | <0.000 | *** |
| Habitat fragmentation | 1.127 | 0.504 | 2.233 | 0.026 | * |
| Habitat loss | 1.178 | 0.431 | 2.732 | 0.006 | ** |
| Habitat preference | 1.035 | 0.355 | 2.912 | 0.004 | ** |
| Habitat structure | 1.275 | 0.561 | 2.271 | 0.023 | * |
| Suitable habitat | -1.718 | 0.277 | -6.204 | <0.000 | *** |
| Habitat type | -0.800 | 0.234 | -3.426 | 0.001 | * |
| Unsuitable habitat | -15.953 | 371.528 | -0.043 | 0.966 |  |
| Micro-habitat | 0.322 | 0.346 | 0.931 | 0.352 |  |
| Natural habitat | 0.641 | 0.545 | 1.175 | 0.240 |  |

Supplementary Table 5. Parameter estimates from a binomial generalized linear model fitted to compare correct use of habitat and related terms between animal-based articles and those including plants for the contemporary data. Note: the correct use of habitat in animal-based articles is the reference category in this analysis. Significance *** P < 0.001, ** P < 0.01, * P < 0.05, n P < 0.1.

|  | Estimate | Std. Error | z value | Pr(>\|z\|) |  |
| --- | --- | --- | --- | --- | --- |
| (Intercept) | 0.269 | 0.134 | 2.003 | 0.045 | * |
| Plant | -1.029 | 0.290 | -3.543 | 0.000 | *** |
| Other terms | -0.213 | 0.239 | -0.888 | 0.374 |  |
| Critical habitat | -0.106 | 0.841 | -0.126 | 0.900 |  |
| Habitat selection | 1.722 | 0.552 | 3.117 | 0.002 | ** |
| Habitat quality | 0.586 | 0.359 | 1.633 | 0.103 |  |
| Habitat use | 0.692 | 0.353 | 1.963 | 0.050 | n |
| Habitat fragmentation | 0.424 | 0.627 | 0.676 | 0.499 |  |
| Habitat loss | 1.209 | 0.466 | 2.595 | 0.009 | ** |
| Habitat preference | 0.785 | 0.416 | 1.888 | 0.059 | n |
| Habitat structure | 2.024 | 0.766 | 2.642 | 0.008 | ** |
| Suitable habitat | -1.606 | 0.378 | -4.246 | 0.000 | *** |
| Habitat type | -0.811 | 0.297 | -2.726 | 0.006 | ** |
| Unsuitable habitat | -15.757 | 415.221 | -0.038 | 0.970 |  |
| Micro-habitat | 0.812 | 0.431 | 1.886 | 0.059 | n |
| Natural habitat | 0.272 | 0.495 | 0.549 | 0.583 |  |

Supplementary Table 6. Parameter estimates from a binomial generalized linear model fitted to compare correct use of habitat and related terms between the top 20 ISI/Google-ranked journals and all other journals for the contemporary data. Note: the correct use of habitat in other journals is the reference category in this analysis. Significance *** P < 0.001, ** P < 0.01, * P < 0.05, n P < 0.1.

|  | Estimate | Std. Error | z value | Pr(>\|z\|) |  |
| --- | --- | --- | --- | --- | --- |
| (Intercept) | 0.357 | 0.154 | 2.317 | 0.021 |  |
| Ranking Top 20 | -0.240 | 0.173 | -1.385 | 0.166 |  |
| Other terms | -0.267 | 0.247 | -1.083 | 0.279 |  |
| Critical habitat | 0.194 | 0.926 | 0.209 | 0.834 |  |
| Habitat selection | 1.674 | 0.554 | 3.021 | 0.003 | ** |
| Habitat quality | 0.573 | 0.388 | 1.478 | 0.140 |  |
| Habitat use | 0.694 | 0.355 | 1.958 | 0.050 | n |
| Habitat fragmentation | 0.357 | 0.630 | 0.566 | 0.571 |  |
| Habitat loss | 1.022 | 0.525 | 1.947 | 0.052 | n |
| Habitat preference | 0.811 | 0.434 | 1.869 | 0.062 | n |
| Habitat structure | 2.535 | 1.041 | 2.436 | 0.015 | * |
| Suitable habitat | -1.512 | 0.384 | -3.935 | <0.000 | *** |
| Habitat type | -0.789 | 0.308 | -2.561 | 0.010 | * |
| Unsuitable habitat | -15.751 | 437.961 | -0.036 | 0.971 |  |
| Micro-habitat | 0.825 | 0.492 | 1.677 | 0.094 | n |
| Natural habitat | 0.770 | 0.601 | 1.281 | 0.200 |  |
